# Improving the differentiation potential of pluripotent stem cells by optimizing culture conditions

**DOI:** 10.1038/s41598-022-18400-8

**Published:** 2022-08-19

**Authors:** Takako Yamamoto, Mao Arita, Hirotaka Kuroda, Takashi Suzuki, Shin Kawamata

**Affiliations:** 1 R&D Center for Cell Therapy, Foundation for Biomedical Research and Innovation, Kobe, Japan; 2 Shimadzu Corp., Kyoto, Japan

**Keywords:** Pluripotent stem cells, Embryonic stem cells, Stem-cell differentiation, Cell proliferation, Pluripotency, Stem cells

## Abstract

Embryoid cells and induced pluripotent stem cells (iPSCs) are pluripotent stem cells (PSCs). They retain differentiation and self-renewal potential. However, the differentiation potential of PSCs can be changed by the culture medium. PSCs retain their differentiation potential when cultured with medium that supports the glycolytic pathway, showing high expression of chromodomain-helicase-DNA-binding protein 7 (CHD7), but lose their differentiation potential with medium that supports mitochondrial function, showing reduced levels of CHD7. Labeling cells by their copy number variant profile revealed that genetically different PSC populations can be cultured by medium selection. Another factor that defines the self-renewal potential of PSCs is culture condition. PSCs form colonies as they grow, and spontaneous differentiation inevitably occurs along the rim of these colonies in areas that lack cell-to-cell contact; because of this, undifferentiated cell populations would diminish if differentiated cells are not removed properly. Seeding cells on a less potent cell-binding material may minimize the inclusion of differentiated cells, exploiting the reduced adhesive properties of differentiated cells. Culturing cells with medium that supports the glycolytic pathway, using CHD7 as a biomarker for differentiation potential, and culturing cells on less sticky material can improve the differentiation potential of already established PSC clones.

## Introduction

Embryonic stem cells (ESCs) and induced pluripotent stem cells (iPSCs) are both types of pluripotent stem cells (PSCs). PSCs have two capabilities: (1) the ability to retain self-renewal potential without limitations on the number of mitotic events or the duration of culture and (2) the potential to differentiate into any cell or tissue type without bias. These two capabilities are distinct but correlated, because spontaneous differentiation inevitably occurs while PSCs are maintained in culture conditions for undifferentiated cells. These spontaneous differentiation events reduce the population size of the self-renewing cells in culture and consequently reduce the differentiation potential of the culture, since differentiated cells would no longer respond to differentiation stimuli in the same way that undifferentiated cells do.

In feeder cell cultures, some colonies inevitably include cobblestone-like cell clusters with an appearance that is distinct from undifferentiated cells. These cobblestone-like cells are differentiated cells, which ideally should be removed from the culture. However, removing differentiated cells completely is not easy during culture or even at the time of passage because cells are passaged in small clumps that may contain differentiated cells. In feeder-free culture, passage is carried out either by single-cell suspension seeding or by the seeding of small cell clumps. In both cases, we need to consider the positioning of cells in the entire colony in terms of cell-to-cell contact. Cells located along the rim of the colony lack cell-to-cell contact at one open end, which might trigger uneven segregation during mitosis and may lead to spontaneous differentiation in these cells as a result. Therefore, when designing a cell culture system for undifferentiated cells that form colonies, we need to consider the frequency of spontaneously differentiated cells in the culture.

PSC differentiation potential is influenced by the culture media used for the maintenance of undifferentiated cells. We previously reported that the expression level of chromodomain-helicase-DNA-binding protein 7 (CHD7) is related to the differentiation potential and growth rate of cells when they are seeded in single-cell suspension^1^. Although the mechanism by which the culture medium can alter CHD7 expression and the relationship between the differentiation potential and growth rate of undifferentiated cells still need to be clarified, the differentiation potential and growth rate of undifferentiated cells is positively correlated^1^.

In addition to the correlation between the differentiation potential and self-renewal potential of PSCs, the heterogeneity of iPSC clones also needs to be considered when determining culture conditions that can be used for standardizing iPSC clones. iPSC clones are generated from various somatic cells having a variety of genetic and epigenetic profiles and are reprogrammed with various methods. This is not the case with ESC clones established from blastocysts. Indeed, the variance in the differentiation potential of iPSC clones requires us to repeat the full set of quality control (QC) tests and preclinical safety tests whenever we change iPSC clones; this is one of the major hurdles in the development of iPSC-based cell therapy. Therefore, culture methods that can maintain differentiation potential and minimize the variance in PSCs, especially among iPSC clones, need to be explored for the safety of PSC-based cell therapy. This study aimed to address these issues.

## Materials and methods

### Cells and cell culture

The following five PSC lines were used: H9 ESCs^2^ (WiCell), KhES-1 cells^3^ (Riken BRC), PFX#9 iPSCs^4^ (FBRI), 201B7 cells^5^ (Riken BRC), and SHh#2 iPSCs^4^ (FBRI). Cells were cultured with hPSC culture medium on mitomycin C-treated SNL76/7 cells (SIM strain embryonic fibroblasts; European Collection of Cell Culture). The hPSC culture medium contained Dulbecco’s modified Eagle’s medium/F12 medium (Sigma, St. Louis, MO, USA) supplemented with 20% Knockout Serum Replacement (Gibco, Grand Island, NY, USA), 2 mM l-glutamine (Nacalai Tesque, Kyoto, Japan), 1% nonessential amino acids (Gibco), 0.1 mM 2-mercaptoethanol (Gibco), and 5 ng/mL fibroblast growth factor-2 (Reprocell). For routine passaging, iPSC colonies were detached with CTK solution (2.5 µg/mL trypsin, 1 mg/mL collagenase IV, 20% KSR, 1 mM CaCl_2_/phosphate-buffered saline [PBS], 70% PBS) and split at a 1:3 ratio every 5–6 days.

H9 ESCs, PFX#9, and SHh#2 iPSCs were cultured with serum-free medium (mTeSR1; Stem Cell Technologies Inc.) on human recombinant laminin 521—(Biolamina) coated dishes. Cells were passaged in clumps using Gentle Cell Dissociation Reagent (Stem Cell Technologies) and split at a 1:50 ratio every 5–6 days.

For single-cell seeding, cells were passaged by seeding a single-cell suspension using TrypLE Select (Thermo Fisher Scientific). Cells in single-cell suspensions were seeded at 1 × 10^5^ cells/well in 6-well plates when cultured with Essential 8 (Es8) medium and 1–3 × 10^5^ cells/well when cultured with Repro FF2 (RFF2) medium (ReproCELL; 4 mL/well). The culture medium was changed every day, and the cells were cultured in an incubator (Model 3110; Thermo Fisher Scientific) at 37 °C in an atmosphere containing 5% CO_2_. Three days after seeding, cells were harvested using TrypLE Select (Thermo Fisher Scientific) for single-cell passaging. Morphologies were observed by phase contrast microscopy (IX81; Olympus). PSCs with a normal karyotype were used in this study.

### Small interfering RNA (siRNA) transfection

All reagents were purchased from Thermo Fisher Scientific, unless otherwise specified. Silencer Select Pre-designed human *CHD7* siRNA (cat. no. 4392420; ID: s31140) and Silencer Select Negative Control No. 2 (cat. no. 4390846) were used in transfection experiments. ESCs (3 × 10^5^) were seeded into each well of a vitronectin (VTN)-N–coated 6-well plate with 4 mL Es8 or RFF2 medium, and *siCHD7* or control siRNA was transfected into the cells using Lipofectamine RNAiMAX in accordance with the manufacturer’s instructions. Briefly, cocktail A (9 µL Lipofectamine RNAiMAX Reagent and 150 µL Opti-MEM Medium) was mixed with cocktail B (3 µL of 10 µM *siCHD7* [30 pmol] or control siRNA [30 pmol] and 150 µL Opti-MEM Medium). The mixture was then incubated for 5 min at room temperature, and 250 µL was used for transfection of ESCs with *siCHD7* (final concentration: 6.25 nM) or control siRNA (final concentration: 6.25 nM) for 24 h. The following day, the medium was changed. Two days after seeding, cells were harvested using TrypLE Select and counted or subjected to reverse transcription quantitative polymerase chain reaction (RT-qPCR). The transduction efficiency of the reagent was assessed by RT-qPCR for detection of *CHD7* mRNA in the transfected cells at the designated time points.

### mRNA transfection

*mCHD7* and control mRNA (mock) were used as described previously^1^. ESCs (3 × 10^5^) were seeded in each well of a VTN-N–coated 6-well plate with 4 mL Es8 or RFF2 medium, and *mCHD7* or control mRNA was transfected into the cells using Lipofectamine Messenger MAX in accordance with the manufacturer’s instructions. Briefly, cocktail A (2.5 µL Lipofectamine Messenger MAX transfection reagent and 125 µL Opti-MEM Medium) was incubated for 10 min at room temperature. Then, cocktail B (2.5 µg *mCHD7* or control mRNA and 125 µL Opti-MEM Medium) was prepared and mixed with cocktail A, followed by a 5-min incubation at room temperature. The mixed cocktail (250 µL) was used for transfection of ESCs with *mCHD7* or control mRNA, and cells were incubated for 24 h. The following day, the medium was changed. Two days after seeding, cells were harvested using TrypLE Select and counted or subjected to RT-qPCR. The transduction efficiency of the reagent was assessed by RT-qPCR for detection of *CHD7* mRNA in the transfected cells at the designated time points.

### Embryoid body (EB) formation assays after transfection with siRNA or mRNA

Cells cultured on VTN-N–coated plates with 4 mL Es8 medium were washed once with PBS (−) 24 h after transfection with either siRNA or mRNA. Cells were then detached using a cell scraper (Iwaki), dissociated by pipetting, transferred into low-attachment 6-well plates (Corning, NY, USA), and cultured with Es6 medium with 10 µM ROCK inhibitor for 1 day and with Es6 medium alone for 3 days for EB formation. The sizes and numbers of EBs derived from PSCs were evaluated on day 3 using a CX5 high-content screening platform (Thermo Fisher).

### JC-1 assay

hPSCs were harvested and dissociated to single cells using TrypLE Select. For the control sample, 1 × 10^6^ cells were suspended in 1 mL warm medium and incubated with 1 µL of 50 mM carbonyl cyanide 3-chlorophenylhydrazone at 37 °C for 5 min and then with 10 µL of 200 µM JC-1 at 37 °C for 15 min. For samples, 1 × 10^6^ cells were suspended in 1 mL warm medium and incubated with 10 µL of 200 µM JC-1 at 37 °C for 15 min. Control samples and test samples were washed twice with warm PBS (−) after incubation.

The cells were then analyzed using a FACS Aria II cell sorter (BD Bioscience), and the data were analyzed using FlowJo software (FlowJo LLC, OR, USA).

### qPCR

The expression of self-renewal factors was determined using RT-qPCR. Total RNA was extracted using the RNeasy Micro Kit (Qiagen, Valencia, CA, USA) according to the manufacturer’s instructions. For RT-qPCR, 500 ng DNase-treated RNA was reverse transcribed into cDNA using a QuantiTect Reverse Transcription Kit (Qiagen). RT-qPCR was then performed in triplicate using TaqMan Fast Advanced Master Mix (Thermo Fisher Scientific) on a StepOnePlus PCR system (Thermo Fisher Scientific). Relative quantification was performed using the 2^−ΔΔCt^ method after normalization to glyceraldehyde-3-phosphate dehydrogenase expression.

### Copy numbers of CHD7

The copy numbers of *CHD7* mRNA were evaluated using droplet digital PCR (Bio-Rad Laboratories, Hercules, CA, USA). Briefly, cDNA was synthesized from 5 ng total RNA extracted from cells cultured with hPSCs or Es8 medium using TaqMan Gene Expression Assays (Hs00215010_m1; Thermo Fisher). The PCR mixture was loaded into a Bio-Rad QX-100 emulsification device, and droplets were formed following the manufacturer’s instructions. cDNA was amplified separately in an Applied Biosystems GeneAmp 9700 Thermocycler. Each 20-µL reaction contained 10 µL droplet digital PCR (ddPCR) Probe Supermix, 1000 nM primers, 250 nM probe, and template cDNA, and PCR was carried out using the following cycling conditions: 10 min at 95 °C, 40 cycles of 30 s denaturation at 94 °C and 60 s of annealing/extension at 53 °C, and a final incubation for 10 min at 98 °C. After cycling, raw fluorescence data for each well were exported from the manufacturer’s software (Bio-Rad QuantaSoft v. 1.2) for analysis.

### Measuring reactive oxygen species (ROS) levels in live cells

Cultured cells were stained with MitoSOX Red Mitochondrial Superoxide Indicator (Thermo Fisher) and CellROX Green Reagent (Thermo Fisher), and microscopic images were captured using several microscopes (IX71 [Olympus] and BZ-X810 [KEYENCE]) for live-cell imaging. Flow cytometry analysis and sorting were conducted with a FACS ARIA II (BD Biosciences).

### Liquid chromatography tandem mass spectrometry (LC–MS/MS)

Pretreated samples were analyzed in multiple reaction monitoring mode using triple quadrupole LC–MS/MS (LCMS-8050) and the Cell Culture Profiling Method Package (Shimadzu Corp). This combination enabled us to simultaneously analyze 95 compounds^6^, including basal medium components and secreted metabolites, within 17 min. For normalization of the measured area ratio, the culture medium was collected every 24 h, and fresh medium was added to the cells. Exponential cell growth curves were generated by plotting the cell number counted at every medium collection. The amount of secreted metabolite from a single cell in 1 h was calculated by dividing the area ratio or micromolar concentration measured by LC–MS/MS by the area defined by the cell number curve for the prior 24 h (area ratio per cell per hour or nmol [2 mL culture volume] per cell per hour).

### EB formation assays

For EB formation assays, 1.8 × 10^5^ cultured PSCs were detached from the plates using a cell scraper (Iwaki), dissociated by pipetting, transferred to low-attachment 6-well plates (Corning), and cultured with Es6 medium with 10 µM RI for 1 day and with Es6 medium alone for 13 days for EB formation. The medium was changed every 3 days. The sizes and numbers of EBs derived from PSCs on days 2 and 14 were measured using a CX5 high-content screening platform (Thermo Fisher). Gene expression levels were determined using RT-qPCR with a TaqMan Scorecard Panel (A15870), and gene expression profiles were determined using a RT-qPCR device (QuantStudio 12 K Flex).

### Assessment of copy number variants (CNVs)

Genomic DNA (gDNA) was extracted using a DNeasy Blood & Tissue Kit (Qiagen) according to the manufacturer’s protocol. Briefly, 250 ng gDNA was digested using the restriction enzyme *Nsp*I. Digested DNA was ligated into the *Nsp*I adapter and amplified via PCR. The PCR products were purified and fragmented with DNase I. The fragmented products were end-labeled with biotin and hybridized to Karyostat HD Arrays (Thermo Fisher Scientific) in a GeneChip Hybridization Oven 645 (Thermo Fisher Scientific) overnight. Arrays were washed and stained using a GeneChip Fluidics Station 450 (Thermo Fisher Scientific) and scanned using a GeneChip Scanner 3000 7G (Thermo Fisher Scientific). Scanned data files were generated using GeneChip Command Console Software and analyzed using Chromosome Analysis Suite v3.2 (ChAS; Thermo Fisher Scientific) considering over 100 kb and at least 50 markers for gains and losses.

## Results

### Correlation between PSC differentiation potential and level of CHD7 expression

**Figure 1:**
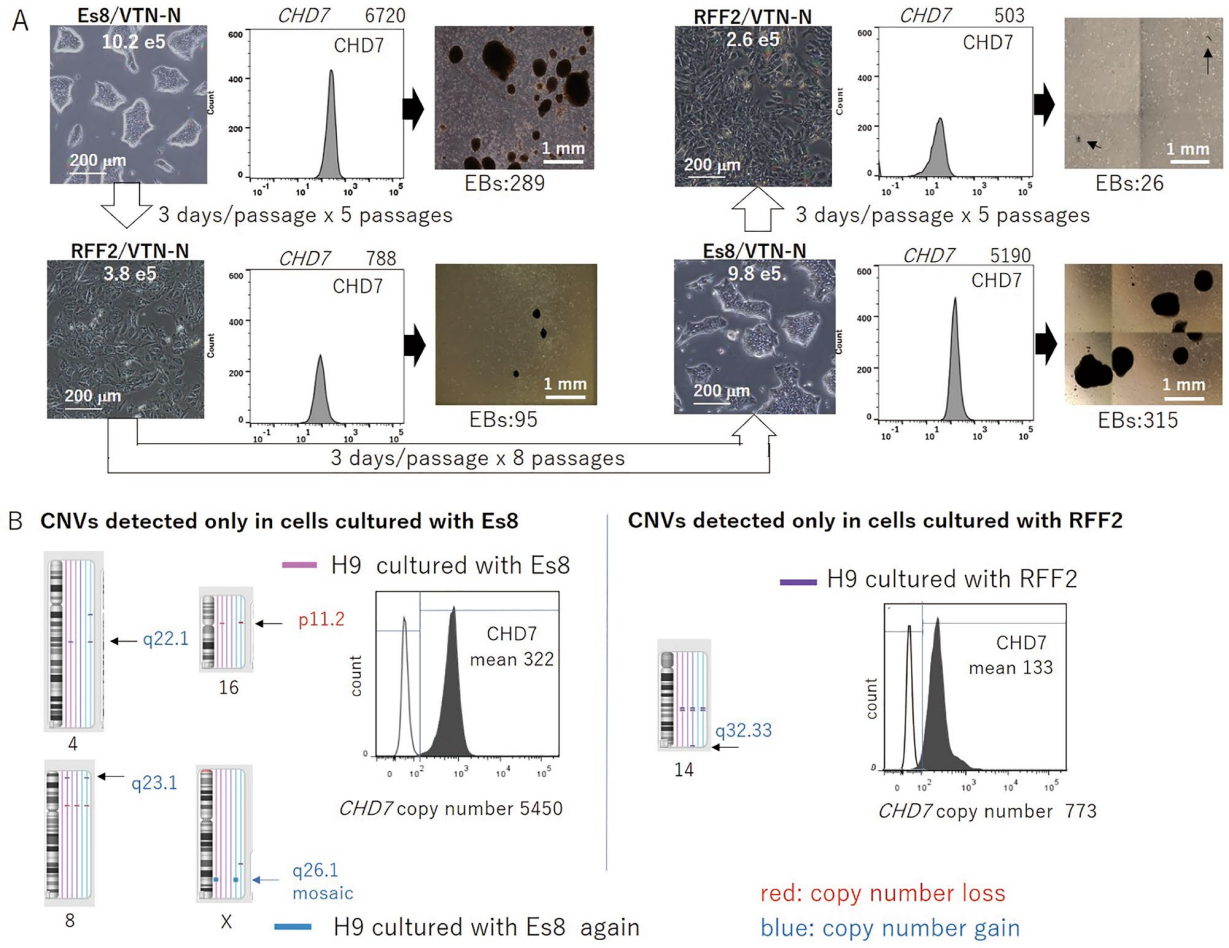
The differentiation potential of cells in culture can be altered by culture medium. (**A**) H9 cells cultured with Essential 8 (Es8) medium on vitronectin-N (VTN)–coated dishes were transferred to RFF2 medium, cultured for 15 days (3 days/passage × 5 passages), transferred again to Es8 medium, cultured 24 days (3 days/passage × 8 passages), and then transferred again to RFF2 medium. Photos of cells in designated culture conditions, with the cell number scored at day 3 after seeding 1.0 × 10^5^ cells (left panels); flow cytometric analysis of CHD7, *CHD7* copy numbers from 5 ng total RNA at day 3 (middle panels); and photographs of EBs formed by day 14 from cells in each culture condition and numbers of EBs formed (right panels). The results are representative of three independent experiments. (**B**) H9 cells were cultured either with Es8 or RFF2 on VTN-N–coated dishes. The loci of copy number variants (CNVs) detected when cells were cultured with Es8 medium (left panels) or RFF2 medium (right panels) are shown. CHD7 expression was determined by flow cytometry (mean values are shown), and *CHD7* copy numbers were determined by digital droplet PCR in cells cultured with Es8 or RFF2 medium.

The potential to differentiate is a critical feature of PSCs used for cell transplantation therapy. Therefore, establishing an assay to evaluate differentiation potential is essential for the maintenance culture of PSCs. EB formation in EB assays is used as a minimum requirement to demonstrate differentiation potential, although EB formation assays may not necessarily guarantee the ability to differentiate into the designated target cells without bias. We used ESC H9 cells in the majority of experiments shown in this study as a representative PSC cell line to minimize the concern of clonal variance in PSC clones that is typically observed among iPSC clones generated from somatic cells with various genetic and epigenetic profiles and with versatile reprogramming methods. H9 cells cultured on VTN-N–coated dishes with Es8 (Thermo Fisher) medium formed a considerable number of EBs; however, the number of EBs was reduced considerably after the cells were transferred to RFF2 medium and cultured for 15 days (3 days/passage × 5). The cells showed an ability to form a comparable number of EBs again when transferred to Es8 and cultured for 24 days (3 days/passage × 8 passages), consistent with our previous report using ESC KhES-1 and iPSC PFX#9^1^. The expression level of CHD7 determined by flow cytometry and the copy number of *CHD7* measured by ddPCR was higher in cells cultured with Es8 than in cells cultured with RFF2 (Fig. 1A). We noted that the cell number scored at day 3 was approximately 3 times higher in cells cultured with Es8 than with RFF2. There was a positive relationship between cell growth rate, CHD7 expression level, and differentiation potential when H9 cells were cultured on VTN-N–coated dishes and passaged in a single-cell suspension.

We next explored the mechanisms through which cells had altered CHD7 expression levels and the ability to form EBs by simply changing the culture medium. There were at least two possible explanations for this mechanism. First, cells in culture might exhibit alterations in both CHD7 expression and the resultant differentiation potential because of signals initiated and mediated by certain factors in the medium. Alternatively, CHD7 expression levels might be genetically and epigenetically predetermined in individual cells and might not be regulated or changed by signals triggered by factors in the culture medium. In the latter case, CHD7 expression levels in cultured cells might change if different dominant cell populations were selected based on a growth advantage in a new culture medium. To evaluate these possible mechanisms, cells in the culture were marked by their CNVs so that changes in the dominant cell population could be detected by comparing CNV profiles. H9 cells cultured with Es8 medium were transferred to RFF2 medium and then were placed back in Es8 medium, and the CNV profiles of H9 cells were examined and compared. Notably, the CNV profiles of cells cultured with Es8 medium included CNVs at loci 4q22.1, 8q23.1, 16p11.2, and Xq26.1, whereas cells cultured with RFF2 medium had CNVs at none of these loci. Additionally, cells cultured with RFF2 medium contained CNVs at the specific locus 14q32.33, and these CNVs were not detected in cells cultured with Es8 medium, indicating that the cell population cultured with Es8 medium was different from that cultured with RFF2 medium (Fig. 1B). This observation led us to explore the mechanisms through which certain cell populations could be selected to expand under specific culture conditions.

### Initiation of differentiation was coupled with the activation of mitochondrial membrane potential

**Figure 2:**
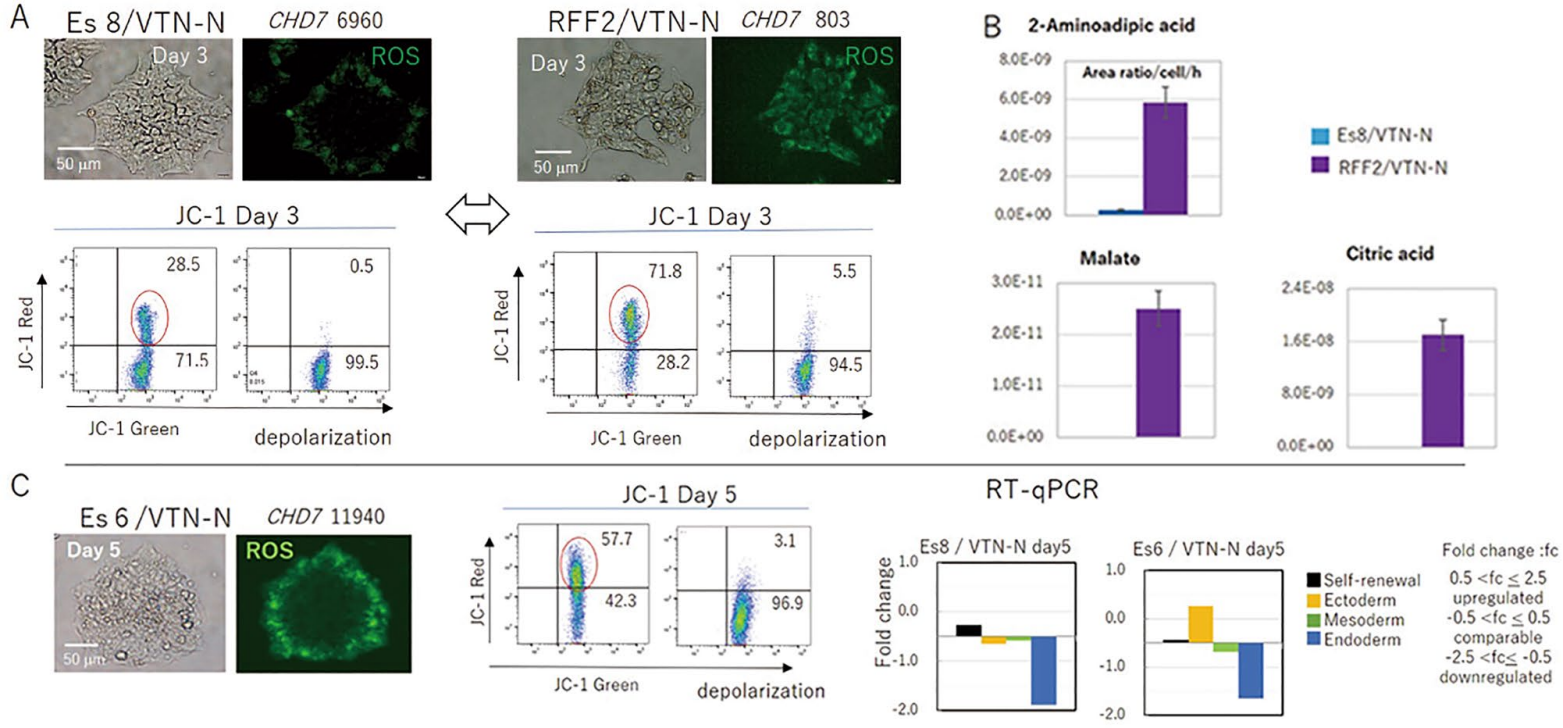
Activation of mitochondrial function is coupled with differentiation. (**A**) Morphology, CellROX (ROX) immunostaining, *CHD7* copy numbers, and mitochondrial membrane voltage (JC-1 assays) in cells cultured with Es8 medium on VTN-N–coated dishes (Es8/VTN) for 3 days (left panels) or with RFF2 medium on VTN-N–coated dishes (RFF2/VTN) for 3 days (right panels) are shown. Mitochondrial membrane voltage was assessed by subtracting baseline electrons (after depolarization) from total electrons (red circle). The percentage of each fraction in the scatter plot of JC-1 assays is shown. (**B**) H9 cells were cultured with Es8 or RFF2 medium, and culture medium was collected and replaced with fresh medium every day for 3 days. 2-Aminoadipic acid (2-AAA), malate, and citrate levels in culture medium were measured using LC–MS/MS. The measured values were standardized as the mean area ratio/cell/h for 3 days. The average values (n = 3) with error bars (SD) are shown in the bar graphs. The results of three independent experiments are shown. (**C**) Morphology, ROX staining, mitochondrial membrane voltage (JC-1 assays; red circle), and gene expression profiles (RT-qPCR score card panels) of H9 cells cultured with Es8 medium on VTN-N–coated dishes on day 5 (left panel: starting material for differentiation by Es6 medium) and Es6 medium on VTN-N–coated dishes on day 5 are shown (right panel). The interpretation of gene expression levels by RT-qPCR is shown in the attached table. The results of three independent experiments are shown.

Next, we explored the impact of cell culture medium on the metabolic systems of cultured cells. The major metabolic pathway used by PSCs and cancer cells is the glycolytic pathway^7^, which is coupled with suppression of mitochondrial activity, as reflected by a low mitochondrial membrane potential (ΔψM) and reduced ROS in the mitochondria^8,9^. We found that the majority of cells cultured with Es8 medium did not show marked ROX staining, which was used to detect ROS produced by mitochondrial activity; the exception was that cells along the rims of colonies did show ROX staining. Furthermore, JC-1 assays showed a suppression of mitochondrial membrane voltage, suggesting that there was no marked mitochondrial activity by day 3 of culture (Fig. 2A). In contrast, cells cultured with RFF2 showed marked ROX staining in most cells and an activated mitochondrial membrane potential by the JC-1 assays, suggesting activated mitochondrial function in cells cultured with RFF2 (Fig. 2A). RFF2 medium contained high concentrations (approximately 23 mg/mL) of protein and various amino acids in addition to moderately high glucose (2.52 g/L), which could support mitochondrial function. However, Es8 medium contained high glucose (3.1 g/L) and a limited amount of amino acids. Thus, Es8 medium could support the glycolytic pathway and at the same time limit the activation of mitochondrial function. The suppressed mitochondrial membrane voltage of cells cultured with Es8 medium supported this idea. There was a reciprocal relationship between the expression of *CHD7* and mitochondrial function when cells were maintained in an undifferentiated state (Fig. 2A). Metabolic analysis showed that the RFF2 culture medium contained malate and citrate as a result of activation of the tricarboxylic acid cycle in cells, whereas the Es8 culture medium did not (Fig. 2B), consistent with the above argument. Furthermore, 2-aminoadipic acid (2-AAA) was detected in the RFF2 medium but not in the Es8 medium (Fig. 2B), indicating that the kynurenine catabolic pathway, which leads to loss of an undifferentiated state and initiation of ectoderm differentiation^6^, was activated in cells cultured with RFF2. This observation suggested that some cells cultured with RFF2 exhibited activated mitochondrial function and underwent spontaneous differentiation, but could not be maintained in RFF2 as this medium lacked the factors necessary to support differentiated cells, and therefore these cells died. Thus, only undifferentiated cells with mitochondrial activation below the permissible level not to undergo differentiation could be cultured and maintained with the RFF2 medium. A positive correlation between the activation of mitochondrial membrane voltage and the initiation of differentiation, as suggested by the secretion of 2-AAA, was observed during the culture of cells with RFF2. This observation was supported by additional experiments; namely, H9 cells cultured with Es6 medium depleted of basic fibroblast growth factor and transforming growth factor β1 compared with Es8 medium showed both an initiation of ectodermal differentiation, as demonstrated by gene expression profiling using RT-qPCR (Fig. 2C, Fig. S1), and an elevated mitochondrial membrane voltage (Fig. 2A,C). Thus, there is evidence that the activation of mitochondrial function is coupled with the initiation of differentiation processes. Next, we examined the impact of elevated CHD7 expression levels and the induction of spontaneous differentiation by introducing *mCHD7* into undifferentiated cells.

### Induction of mCHD7-induced spontaneous differentiation

**Figure 3:**
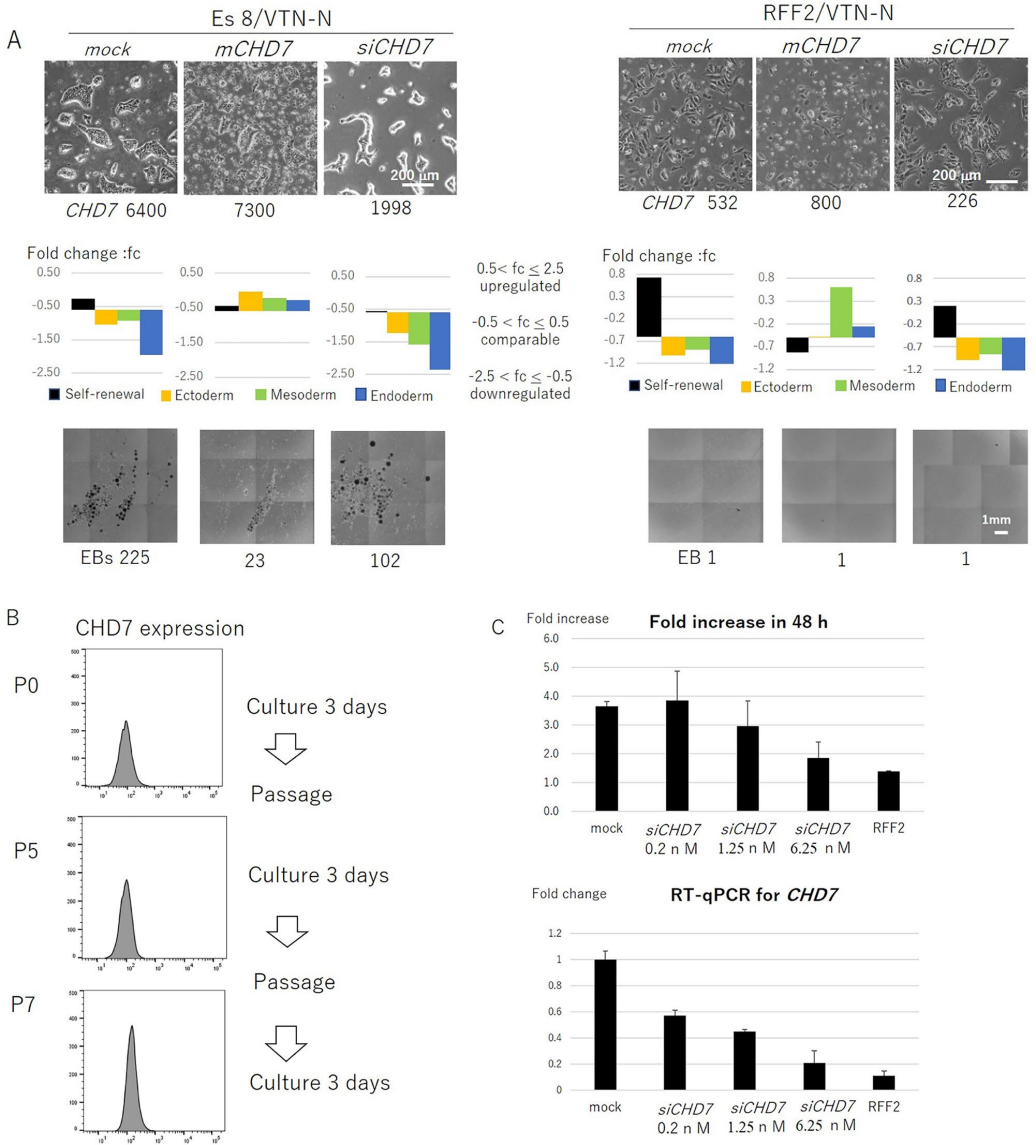
*CHD7* expression affected the differentiation potential and growth of undifferentiated cells. (**A**) H9 cells cultured with Es8 on VTN-N–coated dishes (Es8/VTN, left panels) or with RFF2 on VTN-N–coated dishes (RFF2/VTN, right panels) were transfected with mock (control), *mCHD7*, or *siCHD7*. The morphology, *CHD7* copy numbers, gene expression profiles (RT-qPCR), EB morphology, and EB numbers formed at day 14 under different culture conditions are shown. The representative results of three independent experiments are shown. (**B**) CHD7 expression in H9 cells determined by flow cytometry after cells were transferred from RFF2 to Es8 on VTN-N–coated dishes at passage 0 (P0), P5, and P7. Cells were cultured for 3 days between passages. (**C**) Fold increase of H9 cells after 48 h (upper panel) and *CHD7* expression, as determined by RT-qPCR, after transfection of H9 cells with various doses of *siCHD7* (lower panel). The average values (n = 3) with error bars (SD) are shown in the bar graphs. Representative data from three independent experiments are shown.

There was a positive correlation between the level of CHD7 expression in undifferentiated cells and the differentiation potential manifested by the number of EBs formed in the EB formation assay (Fig. 1A). Interestingly, *mCHD7* induced differentiation of the three germ layers simultaneously, as determined by RT-qPCR in cells cultured with both Es8 and RFF2 media (Fig. 3A, Fig. S2), suggesting a positive role of *CHD7* in both endodermal and mesodermal differentiation processes as well as in ectodermal development. Furthermore, this suggested that there is an upper permissible level of CHD7 being in an undifferentiated state. Es8 and RFF2 media are designed to support the proliferation of undifferentiated cells, not differentiated cells, and cells that forced to differentiate following the introduction of *mCHD7,* could not be maintained in these culture media. Consequently, the number of cells to form EBs was markedly reduced after introduction of *mCHD7* (Fig. 3A). Moreover, the introduction of *siCHD7* reduced the differentiation potential of cells cultured with Es8, as reflected by the marked reduction in the number of EBs formed (Fig. 3A). The introduction of *siCHD7* to cells cultured with RFF2 further reduced the level of *CHD7* and naturally led to no or few EBs being generated. These results provided evidence for the observation in Fig. 1A, demonstrating that the differentiation potential of undifferentiated cells correlated with CHD7 expression.

It is interesting to note that both the increased expression of *mCHD7* and the activation of mitochondrial function induced differentiation. Therefore, there must be a reciprocal relationship between these events in cells in an undifferentiated state. In other words, cells with activated mitochondrial function need to express a limited level of CHD7 to grow in an undifferentiated state at the expense of having a reduced differentiation potential, whereas cells with suppressed mitochondrial function could have relatively high CHD7 levels, enabling these undifferentiated cells to retain differentiation potential. The level of CHD7 that can ensure the differentiation potential of cells varied across cell lines and culture methods, therefore we cannot determine a universal cutoff value for every cell line. However, H9 cells with a *CHD7* copy number of less than 2000 copies/5 ng total RNA showed a limited differentiation potential when cultured on VTN-N–coated dishes (Figs. 1B, 2A, 3A).

### CHD7 expression level correlates with the cell growth rate

In the previous sections, we have shown (1) the introduction of *mCHD7* induced spontaneous differentiation (Fig. 3A), (2) the differentiation process was coupled with the activation of mitochondrial function (Fig. 2C), and (3) there was a reciprocal relationship between the CHD7 expression level and the degree of mitochondrial function in undifferentiated cells (Fig. 2A). The question is how the CHD7 expression and the degree of mitochondrial function corelated each other. We showed culture medium selected a cell population to grow (Fig. 1B), and the activation of mitochondria of cells in culture is directly affected by the formula of culture medium (Fig. 2A). While, we could not demonstrate the relationship between formula of the medium and the expression of CHD7, rather the CHD7 expression level in cells as assessed by flow cytometry showed a broad coefficient of variation (CV) just after the culture medium was changed from RFF2 to Es8 (Fig. 3B, P0). Then, the level of CHD7 expression came to converge at the highest level during the culture (Fig. 3B, P5 and P7). This result suggests that cells with a higher CHD7 expression have a growth advantage and become dominant during the culture. This presumption was manifested by the *CHD7* knockdown experiment using *siCHD7*. This experiment indicated that the level of *CHD7* was positively correlated with cell proliferation potential (Fig. 3C) and cells with a higher CHD7 expression became dominant due to a growth advantage after a couple of passages. This would explain the observation that the expression of CHD7 reached its highest level during the late passages, as shown in Fig. 3B (P7).

### The size of the undifferentiated cell population was affected by the type of extracellular matrix (ECM) used in culture

**Figure 4:**
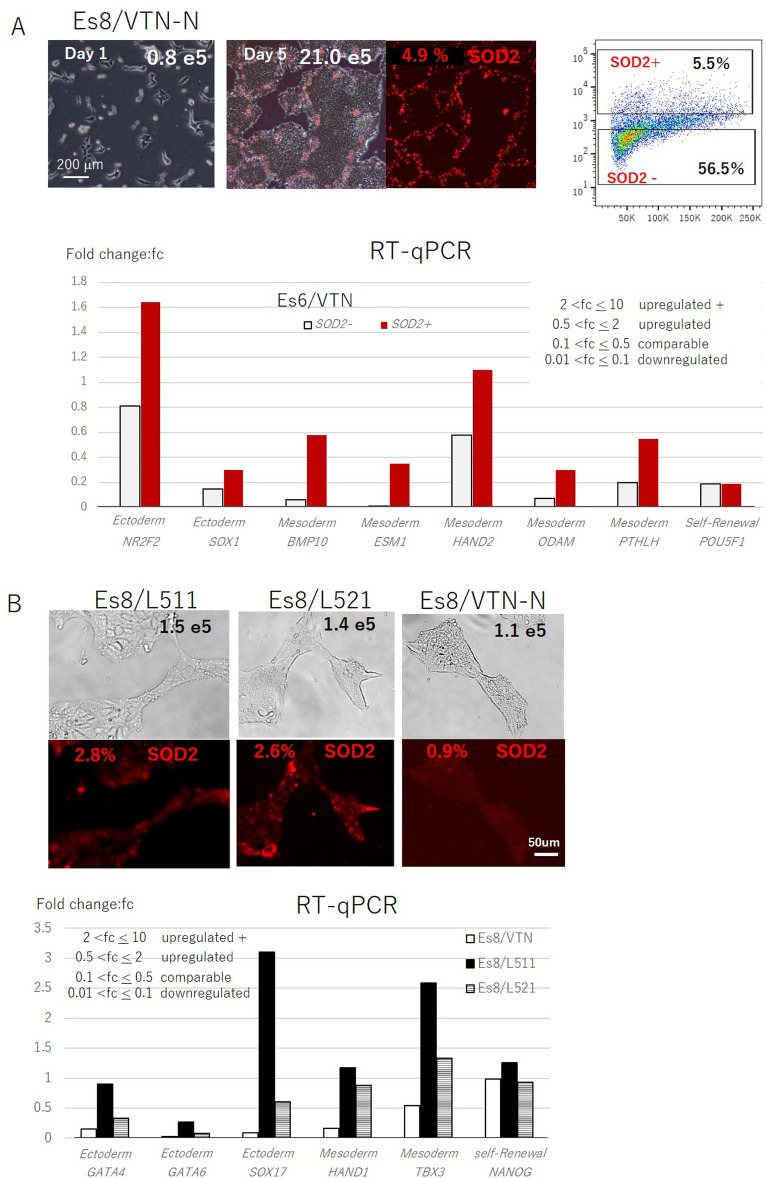
The removal of differentiated cells by seeding on a less adhesive material. (**A**) H9 cells cultured with Es8 on L511-coated dishes for 5 days were stained with anti-SOD2 antibodies (upper left panel), and SOD2-positive (red dots) and SOD2-negative (black dots) cells were sorted (upper right panel) to examine the ectodermal or mesodermal gene expression patterns of each population by RT-qPCR (bottom panel). (**B**) H9 cells cultured with the conditions described in panel A (total 2.1 × 106 cells, 4.9% SOD2-positive cells) were collected and 5.0 × 10^4^ cells from them were seeded as single-cell suspensions either on L511-, L521-, or VTN-N–coated dishes and cultured for 30 h with Es8. The total cell numbers harvested and the percentages of SOD2-positive cells under different culture conditions are shown. The ectodermal or mesodermal gene expression levels of cells cultured under relevant conditions as determined by RT-qPCR are shown in the lower bar graph. The interpretation of gene expression levels determined by RT-qPCR is shown in the attached table. Representative results from three independent experiments are shown.

In addition to the differentiation potential, the retention of self-renewal potential is a key feature of PSCs. PSCs require cell-to-cell contact to grow and, therefore, PSCs need to form colonies. For the clinical application of PSCs, we must focus on an animal-free cell culture system. Therefore, synthetic ECM was used as the dish-coating material based on regulatory considerations. However, cells on the rims of the 2-dimensional (2-D) colonies lack the signals triggered by cell-to-cell contact at one open end, which is in sharp contrast with the majority of cells located in the middle of the colony that are surrounded by other cells along their cell membrane without interruption. Cells along the rim of the colony have an uneven distribution of molecules and ion flux related to the cell-to-cell contact-mediated signals and undergo uneven segregation in mitosis. This, then, results in a break of the self-renewal state where two identical daughter cells are generated from a mother cell, triggering spontaneous differentiation^10–12^. Indeed, cells on the rims of the colonies were positively stained with anti-superoxide dismutase 2 (SOD2) antibodies (Fig. 4A). SOD2 is an enzyme that belongs to the Fe/Mn superoxide dismutase family, which scavenges excess ROS generated as a result of mitochondrial activation. *SOD2* gene expression in H9 cells in the culture showed that these cells committed ectoderm and mesoderm differentiation (Fig. 4A). Consequently, the population of undifferentiated cells would decrease if the spontaneously differentiated cells were not properly removed from the culture. Notably, the percentage of *SOD2*-positive cells (4.9%) on day 5 of culture with Es8/L511 was reduced after cells were seeded in single-cell suspensions on VTN-N–(0.9%), L521-(2.6%), or L511-(2.8%) coated dishes after 30 h (Fig. 4B). This suggests that the ability of cells to adhere to the ECM was reduced in differentiated cells compared with undifferentiated cells, and the cell-binding ability of L511 or L521 for differentiated cells was higher than that of VTN-N. Gene expression profiles showed that cells cultured on L511 or L521 were committed to ectoderm and mesoderm differentiation (Fig. 4B). Thus, by exploiting the reduced cell adhesion properties of differentiated cells and the less potent cell-binding properties of VTN-N, differentiated cells could be effectively eliminated from the culture at a single-cell level by seeding cells in a single-cell suspension at each passage.

### Improvement of the differentiation potential of iPSCs by “recloning” cells using optimized culture conditions

**Figure 5:**
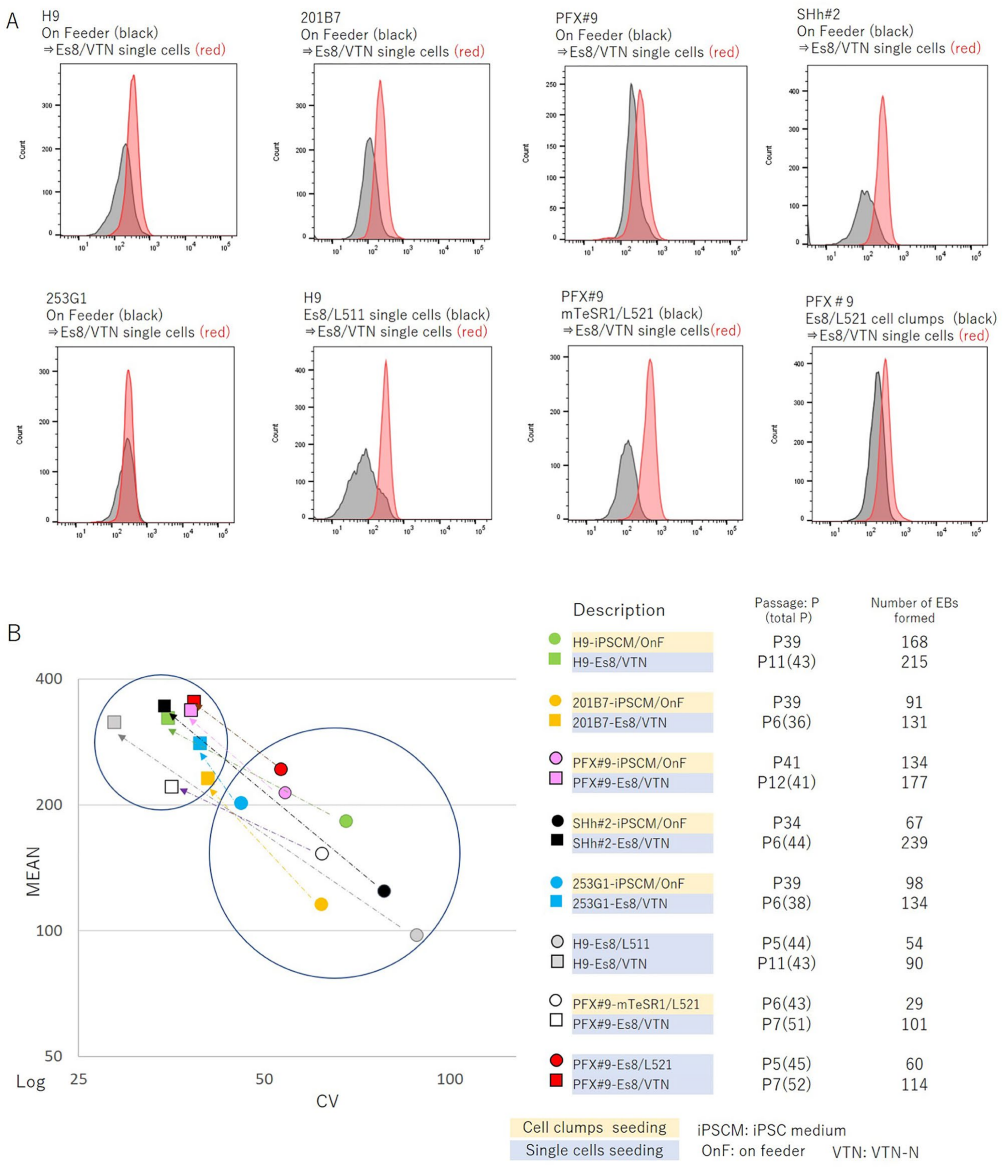
Recloning of cells with differentiation potential based on culture conditions. (**A**) iPSC clones (201B7, PFX#9, SHh#2, or 253G1 cells) or ESC clones (H9 cells) were cultured either on feeder cells or on L511- or L521-coated dishes with iPSC or mTeSR1 medium. Clones were then transferred to Es8 medium and cultured on VTN-N–coated dishes. The mean and convergence of CHD7 expression of cell clones was determined by flow cytometry before (gray histogram) and after (red histogram) changing culture conditions. Representative results from three independent experiments are shown. (**B**) Flow cytometric analysis of cell clones for the mean and coefficient of variation (CV) measured before (circle) and after (square) changing culture conditions are plotted on the left panel and the differentiation potential before and after changing the culture conditions was assessed by the number of EBs formed and is shown on the right panel. The data set shown in (**B**) was generated from the same samples shown in (**A**).

In previous sections, we showed data using ESC H9 cells as the standard control PSC clone to avoid possible arguments about iPSC clones having diverse genetic and epigenetic backgrounds. Therefore, there is a strong need to standardize iPSC clones to develop iPSC-based cell therapy. In the previous section, we showed that the differentiation potential of even ESC H9 cells, which have relatively homogenous genetic and epigenetic profiles, could be altered by culture medium (Fig. 1) and there is a possibility that we can improve the differentiation potential by optimizing culture conditions. Optimized culture conditions may include the selection of an appropriate culture medium that supports the glycolytic pathway, the seeding of cells as single-cell suspensions during passaging, and the culture of cells on an ECM substrate with a relatively weak cell-binding capacity, such as VTN-N, to minimize the inclusion of differentiated cells in undifferentiated cell cultures and to maintain the self-renewal population for the expansion of cell clones. To verify that culture conditions improved the differentiation potential of established iPSC clones, we cultured the iPSC clones 253G1^13^, 201B7^5^, PFX#9, and SHh#2^4^ and the ESC clone H9 (control) with iPSC medium^4^ or mTeSR1 and maintained them on feeder cells or on L511- or L521-coated dishes that were transferred to Es8 medium, cultured on VTN-N–coated dishes, and passaged as single-cell suspensions. The CHD7 expression profile by flow cytometry and the number of EBs formed before and after the transition to Es8/VTN-N culture were measured. Notably, increased levels of CHD7 expression by flow cytometry before and after “recloning” (Fig. 5A) may be a good index for an improved differentiation potential of cells, as manifested by an increase in the number of EBs formed (Fig. 5B). The convergence of CHD7 expression by flow cytometry (Fig. 5A) may represent a decreased variance in the differentiation potential among iPSCs in a given culture.

Although we cannot alter the genetic background of individual cells by changing culture conditions, a cell population with a higher differentiation potential could be selected to grow, or be “recloned,” by culture conditions that support the glycolytic pathway and by eliminating spontaneously differentiated cells by seeding on an ECM with a less potent cell-binding capability, thus exploiting their reduced adhesive properties. This could also reduce the variability in differentiation potential, especially among iPSC clones.

## Discussion

In this study, we showed that the expression level of CHD7 is a good marker for predicting the differentiation potential and monitoring the variance in differentiation potential among PSCs in culture. However, we also found a positive role of CHD7 in initiating and propagating the differentiation process in PSCs. The role of CHD7 in tissue and organ development has been studied elsewhere^14,15^, and mutations in *CHD7* may result in the development of CHARGE syndrome^16^. In addition, we showed that CHD7 was also involved in cell proliferation. Although the mechanisms through which CHD7 mediates the initiation of differentiation and cell cycle progression are unclear, our previous chromatin immunoprecipitation experiments demonstrated that CHD7 is associated with POU5F1, NANOG, and EP300/CBP, but not BRG-1, in euchromatin, and functions to maintain an undifferentiated state^1^. EP300/CBP is known to promote progression through the G_1_/S phase of the cell cycle by transcribing E2Fs and cyclin E, which associate with cyclin-dependent kinase 2 (CDK2) to form the cyclin E/CDK2 complex^17,18^. The association of EP300/CBP with CHD7 in euchromatin may underlie the mechanism of CHD7-mediated cell proliferation in undifferentiated cells. However, reduced expression of the self-renewal molecules POU5F1 and NANOG in the cells initiating differentiation may block the formation of the CHD7/POU5F1/NANOG complex and, in turn, facilitate its association with other chromatin remodeling complexes, such as the BRG-1 complex containing PBAF, thereby mediating ectodermal differentiation via formation of CHD7/BRG-1-PBAF complexes^19^. Furthermore, BRG-1 suppresses cyclin E expression and increases CDK inhibitor expression, thereby blocking the proliferation of cells with self-renewal potential, including cancer cells^20,21^. This suggests that the CHD7/BRG-1 complex may attenuate the function of the CHD7/EP300/CBP complex, which would inhibit the proliferation of cells in a self-renewal state and facilitate the switch from an undifferentiated state to a differentiated state. Thus, we believe that CHD7 may mediate the proliferation of PSCs in a self-renewal state, regulate the transition from an undifferentiated state to a differentiated state, and promote differentiation at multiple stages by switching its chromatin remodeler partners depending on the developmental stage and signals from the environment. Further studies will provide insights into the function of CHD7 at multiple developmental stages and facilitate the improvement of culture systems that support differentiation protocols without differentiation bias.

Another issue affecting the self-renewal potential of PSCs is the size of the undifferentiated cell population in a given culture. Undifferentiated PSCs cannot proliferate in a single suspension form and need cell-to-cell contact and the formation of colonies for their cell growth. Therefore, cells located along the rim of the colony that lack cell-to-cell contract at one open end undergo continuous, spontaneous differentiation as shown in Fig. 4A. Similar spontaneous differentiation events can occur in the cells located on the surface of spheres in 3-dimensional (3-D) culture systems. Although PSCs need to form colonies to grow, it is even more complicated in spherical cultures. We need to control sphere size to avoid generating oxygen or nutrient gradients, which may trigger differentiation, and there is no chance to use the reduced cell adhesive property of differentiated cells to eliminate spontaneously differentiated cells during passage in 3-D conditions. In a 2-D culture system, we explored the cell-binding properties of L511 (a5b1g1), L521 (a5b2g1), and VTN-N (N-terminal), and in subsequent studies, we will examine new ECM substrates, if available. However, an appropriate ECM substrate should show a balance between cell growth and cell-binding capacity; ECM materials with weak cell-binding capacities cannot anchor a sufficient number of undifferentiated cells and cannot deliver potent cell proliferation–related signals to maintain the self-renewing cell population^22^.

In summary, we propose two major culturing methods that can decrease the variance among iPSC clones and increase the differentiation potential of iPSC clones. Namely, culturing cells with medium that supports the glycolytic pathway, rather than mitochondrial activity, using CHD7 expression as a biomarker for the differentiation potential, and minimizing the inclusion of differentiated cells through culturing cells on less sticky material by exploiting the reduced adhesive properties of differentiated cells. We believe that our study not only offers insights for improving the differentiation potential of established iPSC clones, but also opens the discussion for standardizing PSC clones used for cell therapy.

## Supplementary Information

Supplementary Information.

## Data Availability

The data sets used and/or analyzed during the current study are available from the corresponding author on reasonable request.
