# Supplementary material for: Improving the differentiation potential of pluripotent stem cells by optimizing culture conditions
Source: Sci Rep. 2022 Aug 19;12:14147. doi: 10.1038/s41598-022-18400-8 (PMC9391418; doi:10.1038/s41598-022-18400-8)
Supplement: Supplementary file 1 — Supplementary Information. [file 41598_2022_18400_MOESM1_ESM.pdf]

# Supplementary Information

## Improving the differentiation potential of pluripotent stem cells by optimizing culture conditions

Takako Yamamoto, Mao Arita, Hirotaka Kuroda, Takashi Suzuki and Shin Kawamata

# Supplementary Fig. 1 (Fig. S1)

Gene expression profiles of H9 cells cultured with Es8 on VTN-N (Es8/VTN) for 5 days just before transferring to Es6 medium, and cultured with Es6 on VTN-N (Es6/VTN) for 5 days.

| Target Name | Category | Es8 / VTN day5 | Es6 / VTN day5 |
|---|---|---|---|
| CDH9 | Ectoderm | 0.51 | 22.53 |
| COL2A1 | Ectoderm | 2.50 | 0.95 |
| DMBX1 | Ectoderm | 0.30 | 2.11 |
| DRD4 | Ectoderm | 0.14 | 0.29 |
| EN1 | Ectoderm | 0.02 | 0.47 |
| LMX1A | Ectoderm | 0.01 | 12.33 |
| MAP2 | Ectoderm | 5.74 | 8.84 |
| MYO3B | Ectoderm | 0.45 | 1.48 |
| NOS2 | Ectoderm | 0.87 | 0.51 |
| NR2F1/NR2F2 | Ectoderm | 0.00 | 0.04 |
| NR2F2 | Ectoderm | 0.03 | 2.55 |
| OLFM3 | Ectoderm | 0.35 | 1.07 |
| PAPLN | Ectoderm | 0.53 | 0.52 |
| PAX3 | Ectoderm | 0.05 | 1.93 |
| PAX6 | Ectoderm | 0.05 | 20.44 |
| POU4F1 | Ectoderm | 0.48 | 0.63 |
| PRKCA | Ectoderm | 0.87 | 0.35 |
| SDC2 | Ectoderm | 14.60 | 18.24 |
| SOX1 | Ectoderm | 0.79 | 0.25 |
| TRPM8 | Ectoderm | 0.73 | 11.44 |
| WNT1 | Ectoderm | 0.37 | 8.40 |
| ZBTB16 | Ectoderm | 0.35 | 0.35 |

| Target Name | Category | Es8 / VTN day5 | Es6 / VTN day5 |
|---|---|---|---|
| ABCA4 | Mesoderm | 2.20 | 1.97 |
| ALOX15 | Mesoderm | 0.35 | 0.40 |
| BMP10 | Mesoderm | 0.07 | 0.35 |
| CDH5 | Mesoderm | 3.95 | 1.35 |
| CDX2 | Mesoderm | 0.28 | 2.37 |
| COLEC10 | Mesoderm | 2.34 | 0.72 |
| ESM1 | Mesoderm | 0.02 | 0.31 |
| FCN3 | Mesoderm | 3.72 | 0.17 |
| FOXF1 | Mesoderm | 0.50 | 0.70 |
| HAND1 | Mesoderm | 0.03 | 0.86 |
| HAND2 | Mesoderm | 0.19 | 0.36 |
| HEY1 | Mesoderm | 0.91 | 1.31 |
| HOPX | Mesoderm | 0.07 | 1.09 |
| IL6ST | Mesoderm | 0.91 | 0.46 |
| NKX2-5 | Mesoderm | 0.31 | 0.56 |
| ODAM | Mesoderm | 0.51 | 0.76 |
| PDGFRA | Mesoderm | 0.17 | 0.87 |
| PLVAP | Mesoderm | 1.62 | 1.28 |
| RGS4 | Mesoderm | 0.20 | 0.85 |
| SNAI2 | Mesoderm | 0.95 | 0.09 |
| TBX3 | Mesoderm | 0.02 | 0.70 |
| TM4SF1 | Mesoderm | 0.33 | 3.99 |
| FGF4 | Mesendoderm | 0.11 | 0.58 |
| GDF3 | Mesendoderm | 1.18 | 0.68 |
| NPPB | Mesendoderm | 0.02 | 0.00 |
| NR5A2 | Mesendoderm | 45.72 | 8.50 |
| PTHLH | Mesendoderm | 1.08 | 1.16 |
| T | Mesendoderm | 0.00 | 0.00 |

| Target Name | Category | Es8 / VTN day5 | Es6 / VTN day5 |
|---|---|---|---|
| AFP | Endoderm | 0.00 | 0.00 |
| CABP7 | Endoderm | 0.06 | 0.62 |
| CDH20 | Endoderm | 0.22 | 0.97 |
| CLDN1 | Endoderm | 1.46 | 0.41 |
| CPLX2 | Endoderm | 0.15 | 0.35 |
| ELAVL3 | Endoderm | 0.56 | 0.25 |
| EOMES | Endoderm | 0.03 | 0.07 |
| FOXA1 | Endoderm | 0.05 | 0.14 |
| FOXA2 | Endoderm | 0.08 | 0.00 |
| FOXP2 | Endoderm | 0.28 | 0.47 |
| GATA4 | Endoderm | 0.01 | 0.00 |
| GATA6 | Endoderm | 0.00 | 0.01 |
| HHEX | Endoderm | 0.07 | 0.90 |
| HMP19 | Endoderm | 2.59 | 0.51 |
| HNF1B | Endoderm | 0.05 | 0.00 |
| HNF4A | Endoderm | 0.00 | 0.02 |
| KLF5 | Endoderm | 0.48 | 2.40 |
| LEFTY1 | Endoderm | 0.13 | 0.01 |
| LEFTY2 | Endoderm | 0.03 | 0.02 |
| NODAL | Endoderm | 0.03 | 0.00 |
| PHOX2B | Endoderm | 0.03 | 0.02 |
| POU3F3 | Endoderm | 0.06 | 0.08 |
| PRDM1 | Endoderm | 0.20 | 1.58 |
| RXRG | Endoderm | 0.02 | 0.74 |
| SOX17 | Endoderm | 0.01 | 0.01 |
| SST | Endoderm | 0.48 | 0.18 |

| Target Name | Category | Es8 / VTN day5 | Es6 / VTN day5 |
|---|---|---|---|
| CXCL5 | Self-renewal | 23.89 | 0.82 |
| DNMT3B | Self-renewal | 0.30 | 0.34 |
| HESX1 | Self-renewal | 0.16 | 5.90 |
| IDO1 | Self-renewal | 0.82 | 0.94 |
| LCK | Self-renewal | 2.35 | 0.46 |
| NANOG | Self-renewal | 2.40 | 0.38 |
| POU5F1 | Self-renewal | 0.58 | 0.28 |
| SOX2 | Self-renewal | 0.96 | 2.04 |
| TRIM22 | Self-renewal | 17.56 | 3.64 |

**Fold change legend**

| | |
|---|---|
| fc > 100 | Upregulated |
| 10 < fc <= 100 | |
| 2 < fc <= 10 | |
| 0.5 <= fc <= 2 | Comparable |
| 0.1 <= fc < 0.5 | |
| 0.01 <= fc <0.1 | |
| fc < 0.01 | Downregulated |
| omitted | |

Expressions of 96 genes categorized as ectoderm, mesoderm, mesendoderm, endoderm and self-renewal by TaqMan hPSC Scorecard assay are shown in the Table.

# Supplementary Fig. 2 (Fig. S2)

Gene expression profiles of H9 cells transduced with mock, mCHD7 or siCHD7 were cultured with Es 8/VTN-N

| Target Name | Category | *mock* | *mCHD7* | *siCHD7* |
|---|---|---|---|---|
| CDH9 | Ectoderm | 0.34 | 1.69 | 0.06 |
| COL2A1 | Ectoderm | 0.46 | 0.74 | 0.27 |
| DMBX1 | Ectoderm | 0.04 | 0.52 | 0.03 |
| DRD4 | Ectoderm | 0.02 | 0.51 | 0.01 |
| EN1 | Ectoderm | 0.88 | 1.14 | 0.33 |
| LMX1A | Ectoderm | 0.07 | 0.22 | 0.10 |
| MAP2 | Ectoderm | 4.23 | 14.15 | 3.68 |
| MYO3B | Ectoderm | 0.27 | 0.24 | 0.88 |
| NOS2 | Ectoderm | 0.71 | 0.22 | 0.66 |
| NR2F1/NR2F2 | Ectoderm | 0.02 | 5.28 | 0.05 |
| NR2F2 | Ectoderm | 0.08 | 1.00 | 0.07 |
| OLFM3 | Ectoderm | 0.81 | 1.93 | 0.32 |
| PAPLN | Ectoderm | 0.19 | 0.35 | 0.53 |
| PAX3 | Ectoderm | 0.18 | 13.22 | 0.03 |
| PAX6 | Ectoderm | 0.03 | 3.48 | 0.03 |
| POU4F1 | Ectoderm | 0.69 | 13.62 | 0.22 |
| PRKCA | Ectoderm | 0.94 | 1.05 | 0.77 |
| SDC2 | Ectoderm | 5.55 | 23.56 | 4.59 |
| SOX1 | Ectoderm | 0.14 | 0.12 | 0.14 |
| TRPM8 | Ectoderm | 0.61 | 0.48 | 0.34 |
| WNT1 | Ectoderm | 0.04 | 2.19 | 0.03 |
| ZBTB16 | Ectoderm | 0.06 | 0.07 | 0.08 |

| Target Name | Category | *mock* | *mCHD7* | *siCHD7* |
|---|---|---|---|---|
| ABCA4 | Mesoderm | 0.50 | 0.25 | 0.25 |
| ALOX15 | Mesoderm | 0.42 | 0.39 | 0.49 |
| BMP10 | Mesoderm | 0.36 | 0.70 | 0.04 |
| CDH5 | Mesoderm | 5.76 | 22.87 | 2.13 |
| CDX2 | Mesoderm | 0.14 | 0.30 | 0.05 |
| COLEC10 | Mesoderm | 0.61 | 1.20 | 0.59 |
| ESM1 | Mesoderm | 0.36 | 1.09 | 0.01 |
| FCN3 | Mesoderm | 0.74 | 0.23 | 0.56 |
| FOXF1 | Mesoderm | 0.62 | 0.97 | 0.24 |
| HAND1 | Mesoderm | 0.00 | 0.13 | 0.03 |
| HAND2 | Mesoderm | 1.21 | 11.57 | 0.29 |
| HEY1 | Mesoderm | 0.76 | 3.03 | 0.75 |
| HOPX | Mesoderm | 0.25 | 4.80 | 0.23 |
| IL6ST | Mesoderm | 0.76 | 0.94 | 0.53 |
| NKX2-5 | Mesoderm | 0.89 | 2.41 | 0.23 |
| ODAM | Mesoderm | 2.02 | 8.55 | 0.67 |
| PDGFRA | Mesoderm | 0.16 | 0.59 | 0.09 |
| PLVAP | Mesoderm | 0.68 | 0.28 | 0.76 |
| RGS4 | Mesoderm | 0.23 | 0.57 | 0.34 |
| SNAI2 | Mesoderm | 0.26 | 1.82 | 0.27 |
| TBX3 | Mesoderm | 0.01 | 0.14 | 0.04 |
| TM4SF1 | Mesoderm | 0.92 | 2.83 | 0.65 |
| FGF4 | Mesendoderm | 0.12 | 0.52 | 0.30 |
| GDF3 | Mesendoderm | 2.29 | 1.33 | 4.43 |
| NPPB | Mesendoderm | 0.02 | 5.98 | 0.03 |
| NR5A2 | Mesendoderm | 79.00 | 99.35 | 97.38 |
| PTHLH | Mesendoderm | 0.34 | 61.61 | 0.43 |
| T | Mesendoderm | 0.00 | 0.00 | 0.00 |

| Target Name | Category | *mock* | *mCHD7* | *siCHD7* |
|---|---|---|---|---|
| AFP | Endoderm | 0.01 | 0.05 | 0.01 |
| CABP7 | Endoderm | 0.06 | 0.18 | 0.22 |
| CDH20 | Endoderm | 0.15 | 2.92 | 0.30 |
| CLDN1 | Endoderm | 1.59 | 2.60 | 1.25 |
| CPLX2 | Endoderm | 0.48 | 1.02 | 0.62 |
| ELAVL3 | Endoderm | 0.27 | 0.32 | 0.17 |
| EOMES | Endoderm | 0.05 | 1.95 | 0.03 |
| FOXA1 | Endoderm | 0.07 | 7.44 | 0.01 |
| FOXA2 | Endoderm | 4.66 | 0.04 | 1.73 |
| FOXP2 | Endoderm | 0.63 | 2.26 | 0.14 |
| GATA4 | Endoderm | 0.01 | 0.14 | 0.00 |
| GATA6 | Endoderm | 0.01 | 1.95 | 0.00 |
| HHEX | Endoderm | 0.02 | 1.71 | 0.01 |
| HMP19 | Endoderm | 1.01 | 0.31 | 0.89 |
| HNF1B | Endoderm | 0.04 | 0.15 | 0.02 |
| HNF4A | Endoderm | 0.01 | 0.04 | 0.02 |
| KLF5 | Endoderm | 0.34 | 15.86 | 0.36 |
| LEFTY1 | Endoderm | 0.07 | 0.92 | 0.02 |
| LEFTY2 | Endoderm | 0.02 | 1.04 | 0.00 |
| NODAL | Endoderm | 0.03 | 0.07 | 0.01 |
| PHOX2B | Endoderm | 0.02 | 0.65 | 0.02 |
| POU3F3 | Endoderm | 0.03 | 0.31 | 0.04 |
| PRDM1 | Endoderm | 0.05 | 0.54 | 0.04 |
| RXRG | Endoderm | 0.19 | 0.39 | 0.07 |
| SOX17 | Endoderm | 0.00 | 2.88 | 0.00 |
| SST | Endoderm | 0.80 | 0.93 | 0.19 |

| Target Name | Category | *mock* | *mCHD7* | *siCHD7* |
|---|---|---|---|---|
| CXCL5 | Self-renewal | 5.15 | 32.01 | 1.96 |
| DNMT3B | Self-renewal | 0.32 | 0.29 | 0.33 |
| HESX1 | Self-renewal | 0.85 | 0.44 | 0.74 |
| IDO1 | Self-renewal | 0.75 | 0.36 | 1.14 |
| LCK | Self-renewal | 1.95 | 0.97 | 1.35 |
| NANOG | Self-renewal | 0.69 | 1.18 | 0.54 |
| POU5F1 | Self-renewal | 1.26 | 0.57 | 1.21 |
| SOX2 | Self-renewal | 0.97 | 0.86 | 0.59 |
| TRIM22 | Self-renewal | 2.61 | 16.80 | 2.05 |

**Fold change legend**

| | |
|---|---|
| fc > 100 | Upregulated |
| 10 < fc <= 100 | |
| 2 < fc <= 10 | |
| 0.5 <= fc <= 2 | Comparable |
| 0.1 <= fc < 0.5 | |
| 0.01 <= fc <0.1 | |
| fc < 0.01 | Downregulated |
| omitted | |

Expressions of 96 genes categorized as ectoderm, mesoderm, mesendoderm, endoderm and self-renewal by TaqMan hPSC Scorecard assay are shown in the Table.

# Supplementary Fig. 2 (Fig. S2)

Gene expression profiles of H9 cells transduced with mock, mCHD7 or siCHD7 were cultured with RFF2/VTN-N

| Target Name | Category | *mock* | *mCHD7* | *siCHD7* |
|---|---|---|---|---|
| CDH9 | Ectoderm | 0.01 | 0.07 | 0.00 |
| COL2A1 | Ectoderm | 0.13 | 0.17 | 0.05 |
| DMBX1 | Ectoderm | 0.07 | 0.34 | 0.10 |
| DRD4 | Ectoderm | 0.46 | 0.22 | 0.28 |
| EN1 | Ectoderm | 0.29 | 1.08 | 0.30 |
| LMX1A | Ectoderm | 0.06 | 0.10 | 0.05 |
| MAP2 | Ectoderm | 1.53 | 10.66 | 2.43 |
| MYO3B | Ectoderm | 0.70 | 0.76 | 0.48 |
| NOS2 | Ectoderm | 0.78 | 0.94 | 0.72 |
| NR2F1/NR2F2 | Ectoderm | 0.02 | 0.29 | 0.02 |
| NR2F2 | Ectoderm | 0.14 | 0.32 | 0.17 |
| OLFM3 | Ectoderm | 0.36 | 0.93 | 0.61 |
| PAPLN | Ectoderm | 0.34 | 0.14 | 0.16 |
| PAX3 | Ectoderm | 0.15 | 0.47 | 0.16 |
| PAX6 | Ectoderm | 0.06 | 0.35 | 0.11 |
| POU4F1 | Ectoderm | 0.49 | 0.57 | 0.31 |
| PRKCA | Ectoderm | 0.28 | 0.68 | 0.30 |
| SDC2 | Ectoderm | 16.02 | 27.47 | 17.79 |
| SOX1 | Ectoderm | 0.41 | 0.15 | 0.30 |
| TRPM8 | Ectoderm | 0.92 | 0.02 | 1.03 |
| WNT1 | Ectoderm | 0.33 | 3.85 | 0.04 |
| ZBTB16 | Ectoderm | 0.26 | 0.35 | 0.21 |

| Target Name | Category | *mock* | *mCHD7* | *siCHD7* |
|---|---|---|---|---|
| ABCA4 | Mesoderm | 0.49 | 2.23 | 0.29 |
| ALOX15 | Mesoderm | 2.43 | 1.10 | 2.63 |
| BMP10 | Mesoderm | 0.04 | 2.34 | 1.43 |
| CDH5 | Mesoderm | 0.37 | 18.20 | 0.73 |
| CDX2 | Mesoderm | 0.14 | 1.40 | 0.08 |
| COLEC10 | Mesoderm | 0.96 | 1.07 | 0.57 |
| ESM1 | Mesoderm | 0.73 | 0.70 | 0.79 |
| FCN3 | Mesoderm | 0.84 | 0.57 | 0.56 |
| FOXF1 | Mesoderm | 0.63 | 2.44 | 0.69 |
| HAND1 | Mesoderm | 0.02 | 5.63 | 0.06 |
| HAND2 | Mesoderm | 0.22 | 4.44 | 0.34 |
| HEY1 | Mesoderm | 0.81 | 0.65 | 0.96 |
| HOPX | Mesoderm | 3.91 | 1.55 | 0.31 |
| IL6ST | Mesoderm | 0.59 | 4.08 | 0.82 |
| NKX2-5 | Mesoderm | 1.73 | 8.98 | 1.52 |
| ODAM | Mesoderm | 0.04 | 6.38 | 0.35 |
| PDGFRA | Mesoderm | 1.35 | 0.58 | 1.10 |
| PLVAP | Mesoderm | 1.15 | 0.35 | 0.77 |
| RGS4 | Mesoderm | 0.14 | 1.65 | 0.10 |
| SNAI2 | Mesoderm | 0.22 | 0.68 | 0.46 |
| TBX3 | Mesoderm | 0.21 | 4.93 | 0.39 |
| TM4SF1 | Mesoderm | 0.11 | 0.73 | 0.22 |
| FGF4 | Mesendoderm | 14.27 | 3.24 | 3.79 |
| GDF3 | Mesendoderm | 13.73 | 14.41 | 11.49 |
| NPPB | Mesendoderm | 0.45 | 13.67 | 1.13 |
| NR5A2 | Mesendoderm | 96.08 | 159.46 | 105.36 |
| PTHLH | Mesendoderm | 0.46 | 22.26 | 1.06 |
| T | Mesendoderm | 0.00 | 0.00 | 0.00 |

| Target Name | Category | *mock* | *mCHD7* | *siCHD7* |
|---|---|---|---|---|
| AFP | Endoderm | 0.00 | 0.06 | 0.02 |
| CABP7 | Endoderm | 1.05 | 0.71 | 1.02 |
| CDH20 | Endoderm | 0.38 | 1.45 | 0.49 |
| CLDN1 | Endoderm | 1.86 | 5.01 | 2.19 |
| CPLX2 | Endoderm | 1.72 | 0.55 | 2.05 |
| ELAVL3 | Endoderm | 2.93 | 1.09 | 1.26 |
| EOMES | Endoderm | 0.02 | 0.23 | 0.01 |
| FOXA1 | Endoderm | 0.04 | 0.39 | 0.08 |
| FOXA2 | Endoderm | 0.01 | 0.04 | 0.03 |
| FOXP2 | Endoderm | 0.17 | 0.78 | 0.15 |
| GATA4 | Endoderm | 0.02 | 0.11 | 0.02 |
| GATA6 | Endoderm | 0.03 | 1.96 | 0.04 |
| HHEX | Endoderm | 0.24 | 0.40 | 0.07 |
| HMP19 | Endoderm | 1.48 | 0.65 | 1.07 |
| HNF1B | Endoderm | 0.01 | 0.08 | 0.01 |
| HNF4A | Endoderm | 0.02 | 0.56 | 0.00 |
| KLF5 | Endoderm | 1.37 | 9.88 | 2.04 |
| LEFTY1 | Endoderm | 0.75 | 0.93 | 0.54 |
| LEFTY2 | Endoderm | 0.21 | 0.59 | 0.26 |
| NODAL | Endoderm | 0.98 | 1.29 | 1.26 |
| PHOX2B | Endoderm | 0.02 | 0.03 | 0.02 |
| POU3F3 | Endoderm | 0.03 | 0.12 | 0.03 |
| PRDM1 | Endoderm | 0.15 | 0.85 | 0.16 |
| RXRG | Endoderm | 0.02 | 2.05 | 0.01 |
| SOX17 | Endoderm | 0.05 | 0.47 | 0.00 |
| SST | Endoderm | 4.63 | 11.65 | 5.67 |

| Target Name | Category | *mock* | *mCHD7* | *siCHD7* |
|---|---|---|---|---|
| CXCL5 | Self-renewal | 9.06 | 17.59 | 14.54 |
| DNMT3B | Self-renewal | 1.51 | 0.54 | 1.29 |
| HESX1 | Self-renewal | 0.24 | 0.16 | 0.27 |
| IDO1 | Self-renewal | 1.09 | 0.17 | 0.65 |
| LCK | Self-renewal | 1.13 | 0.74 | 0.88 |
| NANOG | Self-renewal | 2.77 | 2.12 | 2.03 |
| POU5F1 | Self-renewal | 2.83 | 1.11 | 1.59 |
| SOX2 | Self-renewal | 0.00 | 0.05 | 0.42 |
| TRIM22 | Self-renewal | 10.81 | 30.29 | 8.67 |

**Fold change legend**

| | |
|---|---|
| fc > 100 | Upregulated |
| 10 < fc <= 100 | |
| 2 < fc <= 10 | |
| 0.5 <= fc <= 2 | Comparable |
| 0.1 <= fc < 0.5 | |
| 0.01 <= fc <0.1 | |
| fc < 0.01 | Downregulated |
| omitted | |

Expressions of 96 genes categorized as ectoderm, mesoderm, mesendoderm, endoderm and self-renewal by TaqMan hPSC Scorecard assay are shown in the Table.
